# The Effect of Mixed Reality Technologies for Falls Prevention Among Older Adults: Systematic Review and Meta-analysis

**DOI:** 10.2196/27972

**Published:** 2021-06-30

**Authors:** Anna Nishchyk, Weiqin Chen, Are Hugo Pripp, Astrid Bergland

**Affiliations:** 1 Department of Computer Science Faculty of Technology, Art and Design Oslo Metropolitan University Oslo Norway; 2 Faculty of Health Sciences Oslo Metropolitan University Oslo Norway

**Keywords:** falls, fall prevention, mixed reality, augmented reality, virtual reality, physical exercise

## Abstract

**Background:**

Falling is one of the most common and serious age-related issues, and falls can significantly impair the quality of life of older adults. Approximately one-third of people over 65 experience a fall annually. Previous research has shown that physical exercise could help reduce falls among older adults and improve their health. However, older adults often find it challenging to follow and adhere to physical exercise programs. Interventions using mixed reality (MR) technology could help address these issues. MR combines artificial augmented computer-generated elements with the real world. It has frequently been used for training and rehabilitation purposes.

**Objective:**

The aim of this systematic literature review and meta-analysis was to investigate the use of the full spectrum of MR technologies for fall prevention intervention and summarize evidence of the effectiveness of this approach.

**Methods:**

In our qualitative synthesis, we analyzed a number of features of the selected studies, including aim, type of exercise, technology used for intervention, study sample size, participant demographics and history of falls, study design, involvement of health professionals or caregivers, duration and frequency of the intervention, study outcome measures, and results of the study. To systematically assess the results of the selected studies and identify the common effect of MR interventions, a meta-analysis was performed.

**Results:**

Seven databases were searched, and the initial search yielded 5838 results. With the considered inclusion and exclusion criteria, 21 studies were included in the qualitative synthesis and 12 were included in meta-analysis. The majority of studies demonstrated a positive effect of an MR intervention on fall risk factors among older participants. The meta-analysis demonstrated a statistically significant difference in Berg Balance Scale score between the intervention and control groups (ES: 0.564; 95% CI 0.246-0.882; *P*<.001) with heterogeneity statistics of I2=54.9% and Q=17.74 (*P*=.02), and a statistical difference in Timed Up and Go test scores between the intervention and control groups (ES: 0.318; 95% CI 0.025-0.662; *P*<.001) with heterogeneity statistics of I2=77.6% and Q=44.63 (*P*<.001). The corresponding funnel plot and the Egger test for small-study effects (*P*=.76 and *P*=.11 for Berg Balance Scale and Timed Up and Go, respectively) indicate that a minor publication bias in the studies might be present in the Berg Balance Scale results.

**Conclusions:**

The literature review and meta-analysis demonstrate that the use of MR interventions can have a positive effect on physical functions in the elderly. MR has the potential to help older users perform physical exercises that could improve their health conditions. However, more research on the effect of MR fall prevention interventions should be conducted with special focus given to MR usability issues.

## Introduction

### Overview

The world population is rapidly aging. According to the World Population Ageing 2017 report [1], this tendency could be one of the most important changes in modern society and would accordingly have a significant influence on many aspects of our lives. Unfortunately, about one-third of community-dwelling older people over the age of 65 experience falls annually [2,3]. Fall-related injuries, such as hip fractures, are major contributors to the burden of disease in older adults and represent a significant public health problem [4-6]. Reducing falls has become an international health priority [7].

The first step in the development of effective fall prevention methodologies is identifying fall risk factors [8]. Studies have identified risk factors associated with falling among older adults, which include a history of falls, gait problems, vertigo, weakness, unsteady gait, confusion, reduced mobility, heart dysfunction, and functional impairment [9-11].

Physical activity and exercise have been found to be effective for reducing risk factors and the number of falls [10,12,13]. Regular physical training can prevent serious fall-related injuries [14]; enhance mobility; improve aspects of the health of elderly individuals, including their strength and balance; and reduce mortality [15]. However, the effectiveness of evidence-based fall prevention methods is only as strong as the level of adherence to these recommendations [16,17]. Commonly reported average uptake rates for simple exercise interventions among community samples are as low as 10% [4,5,14,18]. A number of researchers have investigated barriers to regular physical activities among older adults and identified various reasons for lack of training, including fear of falling, environmental inconvenience, intimidation by fitness facilities or exercise settings, boredom, and lack of motivation [19-21].

Many studies have addressed the effect of different technological interventions on users’ motivation to perform physical exercises. The results of such studies have demonstrated the effectiveness of interactive interventions such as exergames, virtual reality (VR), and augmented reality (AR). Several researchers [22-24] have highlighted that games help motivate users to perform targeted actions, including physical exercise, and improve their performance in these actions. A previous study [19] has demonstrated a similar effect among older people, highlighting the improved adherence of elderly participants to exercise tasks and the potential fall prevention impact. Another study has demonstrated the positive attitude of older participants toward the use of a fall prevention exercise program and its augmentation with VR intervention [25]. According to Mirelman et al [26], VR is one of the most effective interventions for fall prevention in older people.

A recent systematic literature review investigated the effect of VR interventions on mobility skills and balance measurement improvement [27]. However, it included studies which only used the term “virtual reality” and not the terms “augmented reality” and “mixed reality” (MR), both of which are closely related to VR. In fact, the terms VR and AR are often used to refer to similar or even identical technologies. For instance, Ku et al [28] used a Kinect motion sensor (Microsoft Corporation), a laptop, and a big screen to create an interactive training setup and described this system with the term “augmented reality.” By contrast, Mirelman et al [29] used a similar set up with a modified Kinect sensor, a computer, and a large screen but instead used the term “virtual reality” to describe the system.

Because the concepts of AR and VR are so closely related, Milgram et al [30] coined the term “reality–virtuality continuum” to describe them together as parts of a framework. This framework represents a spectrum of environment types, ranging from solely real to solely virtual, with different ratios of real-world and computer-generated elements in between, essentially describing the many types of “mixtures” that can be implemented in MR. A truly complete investigation into the effectiveness of the entire spectrum of such systems thus must necessarily include variations of all 3 terms—VR, AR, and MR—in a systematic search.

To that end, the aim of this systematic review and meta-analysis is to investigate the use of MR technologies spanning the full spectrum of the reality–virtuality continuum for fall prevention and evaluate existing evidence of its effectiveness. The study provides the results of extensive evaluation of the types of study designs that have been typically used in this area of research and demonstrates the quantitative comparison of the most common outcome measures.

### Background

#### Virtual Reality, Augmented Reality, and Mixed Reality

When it comes to research involving different “reality” technologies, one of the most important issues is lack of understanding of the relevant terminology [31]. The 3 main related terms that are currently most commonly used are VR, AR, and MR [31]. Different studies use different definitions of those technologies, and some studies do not provide any definition of the investigated technologies at all, which complicates any systematic review or analysis of these studies.

According to Höllerer and Feiner [32], VR involves the user interacting with an artificial environment by sight, sound, touch, and the other senses, whereas AR allows the user to experience the real world with additional interactive artificial elements [32].

**Figure 1:**
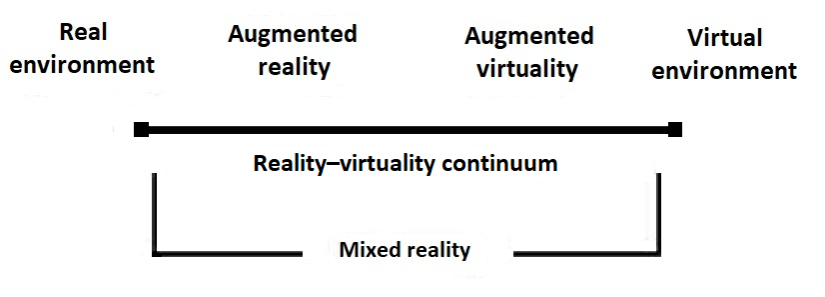
Reality–virtuality continuum framework [32].

Therefore, both VR and AR to some extent combine a virtual world or its artificial augmented computer-generated elements with the real world. Based on common characteristics of the terms, Milgram et al [30] united the concepts under the term MR within a framework they called the reality–virtuality continuum (Figure 1).

In this paper, the term MR will be used in accordance with the framework by Milgram et al [30]. Thus, all studies that use the terms VR, AR, or MR were included in the search procedure.

#### Mixed Reality and Fall Prevention

MR technologies have frequently been used for training and rehabilitation purposes [33]. The popularity of these technologies has drastically increased in recent years, and they have received a lot of attention in scientific research. Many recent papers have discussed the advantages of MR technologies for training.

One of the advantages of using MR for training is that they promote an *external focus of attention* for the user by providing virtual cues (eg, projection onto the floor or a wall) [33]. Motor learning literature divides training instructions into 2 categories: those that promote an internal focus of attention (by referring to the individual’s body parts) and those that promote an external focus of attention (by referring to external objects such as a step box or wall). Recent studies showed that having an external focus of attention results in better motor learning.

Another advantage of MR is that it provides *implicit learning*, which means “learning without awareness of what is being learned” [33]. Implicit learning methods have a better effect on rehabilitation and training than learning with explicit instructions. MR can help to provide an implicit learning effect by creating an external focus of attention, as mentioned above, or by providing a concurrent cognitive task or task variation [33].

MR also helps users to *learn and memorize the physical exercises* better than traditional video demonstration. In a study on the implementation of the AR training system YouMove, Anderson et al [34] demonstrated that this system helps users learn and memorize physical exercises twice as effectively as a conventional video demonstration.

One very important advantage of MR is its ability to *increase users’ motivation* to exercise [33] and, consequently, to help them achieve a *higher exercise intensity*. MR also creates a *contextual situational experience* for the user [35]. MR applications display instructions step by step, identify items, and provide textual information. MR can also create different *exercise variations* and provide *task-specific training* [33].

Specifically regarding elderly users, MR can help older adults perform physical *exercises without trainer supervision*. Kojima et al [15] presented an AR system that provides instructions and control speed of exercises to reduce the risk of physical overload and allow older adults to use it without a supervision. Furthermore, older users have described the use of MR systems as *intuitive and satisfying* [35].

## Methods

### Study Design

This systematic literature review was conducted according to the Preferred Reporting Items for Systematic Reviews and Meta-Analyses (PRISMA) guidelines [36]. The current review methodology consists of 4 main stages: planning, a database search, screening of the results, and analysis of the selected studies. Three researchers (AN, WC, and AB) were involved in the search, screening, data extraction, and analysis processes.

### Planning of the Systematic Literature Review

#### Research Questions

Before conducting the systematic literature review, a preliminary literature review was performed to identify the needs for the systematic literature review. From these preliminary results, the following research questions for the systematic literature review were formulated.

RQ1: Which MR technologies have been used for elderly training and how?

RQ2: How effective are MR technologies interventions in reducing fall risk factors?

#### Definition of “Older”

Different sources provide different estimations of who qualifies as “older” adults, often due to cultural differences and country-specific conditions. For instance, the World Health Organization [37] considers “older adults” to include people of at least age 65, whereas the American Association of Retired Persons uses the term “older adults” to refer to people of age 50 and up [38].

Several relevant studies have used the age group 50 and above as their target group [39-41]. Therefore, we decided to include all studies that focus on people aged 50 years and above to cover all relevant studies.

#### Inclusion and Exclusion Criteria

Because we aimed to cover as many related studies as possible, at this stage of the review, we decided to include both studies on participants with a history of falls and those on elderly participants with a risk of falling.

Based on the above aims, the research questions, and considerations, the following inclusion and exclusion criteria were defined:

- The target group of each study must be people aged 50 and above.
- The study’s participants should not have a chronic disease.
- The study must focus on fall prevention and use physical exercise as a fall prevention method.
- The study must investigate MR intervention.
- The study must include an investigation of the intervention effect, which is measured by the number of falls or risk factors for falling.
- The study must include more than 1 session of physical training.
- The paper must be a scientific publication published in peer-reviewed journal.

We excluded publications such as theses, dissertations, review papers, surveys, preliminary or pilot studies; publications that describe only research design; non-English papers; and research with no control group.

### Database Search

Before the start of the database search, the main search terms were identified based on the research aim and research questions. The main terms, which had to be included in some form in the considered publications, were elderly, falls, and MR technologies. We then also included synonyms and similar terms as alternatives to these main terms, such as “older,” “seniors,” “aged,” and “ageing” for elderly; “fall,” “falls,” and “falling” for falls. In the databases that support the wildcard symbol “*” the search term “fall*” was used. To include all the MR technologies, all 3 discussed above (ie, “augmented reality,” “virtual reality,” and “mixed reality”) were used.

The final generated search string was thus as follows: *(elderly OR older OR seniors OR aged OR ageing) AND (fall OR falls OR falling) AND (“virtual reality” OR “augmented reality” OR “mixed reality”)*

The search was performed iteratively from February 16, 2020, to June 23, 2020. A total of 7 databases were searched: ScienceDirect, Web of Science, ACM Digital Library, IEEE Xplore Digital Library, PubMed, MedlinePlus, and Cochrane Library. The search results in each database are presented in Table 1.

**Table 1:** Number of search results in the electronic databases.

Library	Total number of search results
ScienceDirect	3496
Web of Science	245
ACM Digital Library	363
IEEE Xplore Digital Library	1498
PubMed	134
MedlinePlus	4
Cochrane Library	98
Sum	5838

### Screening Process

The total number of identified results was 5838. After duplicates were eliminated, 5164 records remained, and these were screened for title relevance. At this stage, reviews and dissertations were also removed. The number of excluded studies after title screening was 4751. The abstracts of the remaining 413 papers were read and analyzed. The studies that did not satisfy the inclusion and exclusion criteria were identified. For instance, 54 studies did not consider MR intervention, and 66 did not focus on physical exercises. Detailed explanations of why papers were excluded are presented in Textbox 1. From the 413 papers whose abstracts were analyzed, 331 were excluded at this stage. As a result, 82 papers were assessed and read in full.

At this stage, papers were excluded for the following reasons:

- The authors were not using MR technology as an intervention.
- The focus of the paper was not on physical exercises.
- The study investigated the attitude of the participants toward the technology rather than the effect of the intervention.
- The study had only 1 session of physical exercise.
- The paper described a research design.
- The study did not have a control group.
- The paper presented a preliminary or a pilot study.

**Reasons for study exclusion.**

**1. Papers after title screening (n=413)**

- Focus is not relevant (focus on fear, emotions, cognitive training, learning, disease; n=149)
- No MR intervention (n=54)
- The target group is not the elderly (n=44)
- Survey or review papers (n=6)
- No physical exercise or no fall prevention (n=66)
- Registered trials (no academic publication) (n=10)
- Non-English papers (n=2)

**2. Papers after abstract screening (n=82)**

- Investigate usability, acceptability, or experience (n=9)
- Focus other than fall prevention (n=5)
- Preliminary or pilot studies (n=10)
- Assessment of an evaluation model, algorithm, or measurement system (n=6)
- Study protocol or study design (n=3)
- Include participants who are frail or after trauma (n=11)
- Development or technical investigation (n=4)
- No control group (n=11)
- Conference publications (n=2)

3. Papers after full-text screening (n=21)

**Figure 2:**
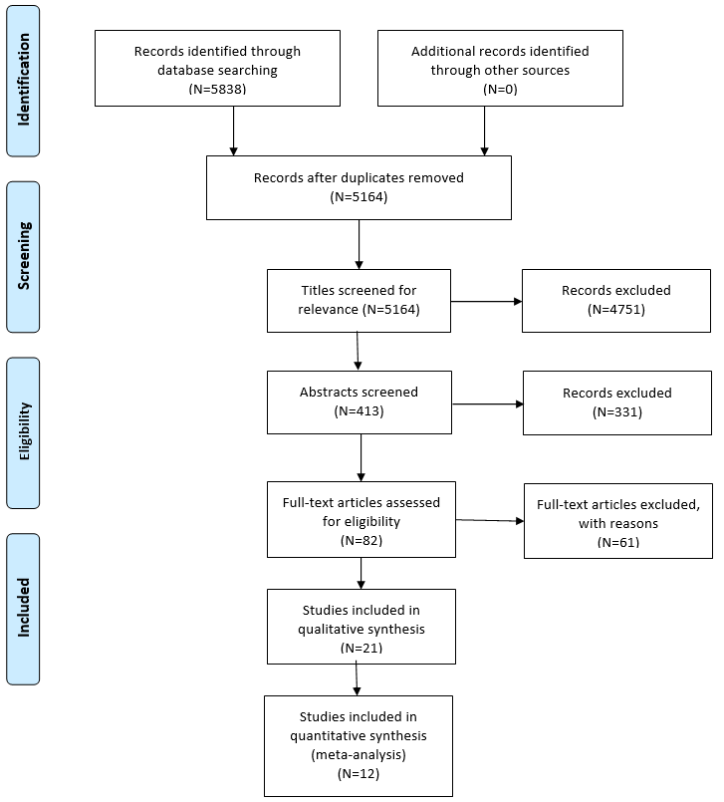
Process of the systematic literature review using a PRISMA flow diagram. PRISMA: Preferred Reporting Items for Systematic Reviews and Meta-Analyses.

After the full-text assessment, 61 more studies were excluded. The remaining 21 papers passed all of the inclusion criteria and were included in the qualitative synthesis (Figure 2). The 12 studies that used the 2 most common outcome measures, namely, the Berg Balance Scale (BBS) [42] and the Timed Up and Go (TUG) test [43], were included in the quantitative synthesis (meta-analysis).

### Qualitative Synthesis

The 21 papers included in the qualitative synthesis were reviewed in detail, with particular focus on the following data, which were extracted and compiled in a data table.

- Aim of the study
- Type of exercise (eg, walking, balance exercise, strength exercise)
- Intervention technology
- Study sample size, demographics, and fall histories
- Study design
- Involvement of health care professionals/caregivers
- Duration and frequency of the intervention; follow-up after the experiment
- Outcome measures
- Results

### Quantitative Analysis

To systematically access the results of the selected studies, identify a common effect of MR intervention, and try to find a relation between different study designs and their results, a meta-analysis was performed. The BBS [42] and TUG [43] scores were the most common outcome measures used in the selected studies with a similar study design. Therefore, studies that used the BBS and TUG were included in the meta-analysis. Other outcome measures were used in too few studies to be included in the meta-analysis.

Because both the BBS [42] and TUG [43] measures were assessed, the standardized mean difference (SMD) between the intervention and control groups were calculated, with a high SMD corresponding to better physical performance with the intervention. Study heterogeneity was assessed by examining the data extraction tables and estimating the *I*^2^ statistic and the Cochran Q test statistic. Based on this, a random effects meta-analysis was considered appropriate. The SMD was calculated with 95% CIs and *P* values to assess the pooled differences between the control and treatment groups within the BBS and TUG measures. Publication bias was assessed using funnel plots and Egger test for the small-study effect. To estimate an overall pooled effect from the BBS and TUG measures, we used a method for robust variance estimation in meta-regression with dependent effect size (ES) estimates implemented in the user-developed Stata package robumeta. This method takes into account dependent effects due to correlated estimation errors when the 2 outcome measures were collected on each participant in several of the studies. For the correlated effects model, the value of the correlation rho was set to 0.8. Stata version 15 (StataCorp) with the user-developed packages metan, metafunnel, metabias, and robumeta was used for all estimations.

## Results

### Qualitative Synthesis of the Selected Studies

#### Overview of Selected Studies

All of the selected studies were published between 2012 and 2020. The papers reported research with similar aims to evaluate the effects of MR intervention on fall prevention for older adults. All of the studies focused on determining the effect of particular MR interventions. Despite the similar goals and methods (12 studies used randomized controlled trials, while 9 were intervention studies with experimental and control groups), the study designs differed in many aspects.

#### Sample Size and Demographics

**Figure 3:**
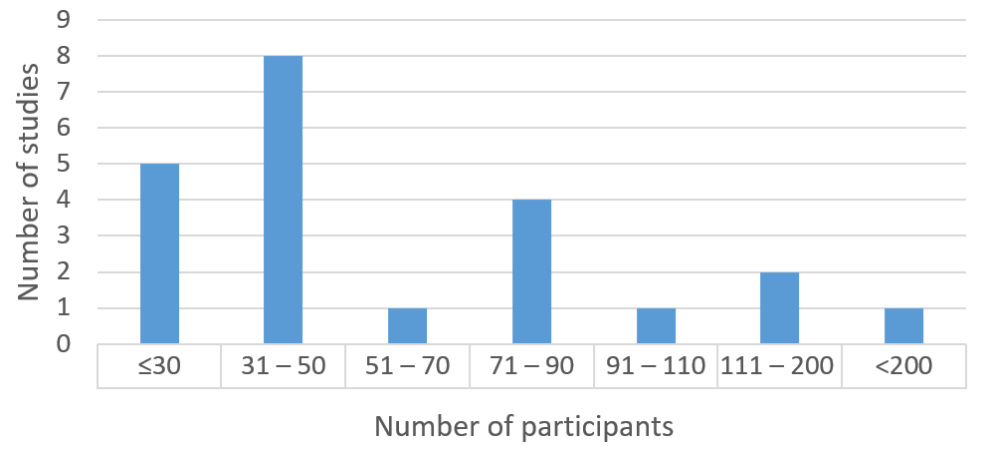
Distribution of number of participants in the selected studies.

The total number of participants over all considered studies was 1524. The number of participants per study ranged from 18 [44] to 302 [29]. The mean number of participants was 72.57 (SD 68.93). A histogram of the number of participants in each study is presented in Figure 3. Of the 21 considered studies, 13 used at least 40 participants (61.9% of studies).

The studies had varying minimum age requirements for participants. Ten studies used 65 years as the minimum age requirement for participation in their study [44-53], and 4 used 60 years [29,54-56]. One study [57] used 66 as the minimum age requirement for participants, and 3 studies [28,58,59] used 55 or 56 years as the minimum. Three more studies did not specify a minimum age requirement for participants [60-62]. The study with the highest mean age was that by Rendon et al [56] with 85.7 (SD 4.3) years for the experimental group and 83.3 (SD 6.2) years for the control group. The study with the lowest mean participant age was that by Singh et al [58] with 61.12 (SD 3.72) years for the experimental group and 64.00 (SD 5.88) years for the control group. The majority of studies recruited participants of both genders; however, 4 focused on older women [53,58,61,62].

Twelve studies [28,29,45,46,48,50-52,54-56,60] involved the recruitment of community-dwelling older people. Yeşilyaprak et al [44] and Htut et al [47] recruited older adults living in nursing or elderly homes; other studies did not specify this choice.

Another participant characteristic considered was a previous history of falling. Four studies [29,44-46] recruited older adults who have experienced a fall before (or had balance issues); the remaining 17 papers did not specify the participants’ history of falls or if they were at a risk of falling.

#### Study Design

Twelve papers [28,29,47,48,50,52,54,56,58-61] described randomized controlled trials, and the remaining 9 reported intervention studies with experimental and control groups and preintervention and postintervention measures of different physical and mental parameters of the participants (Table 2 and Multimedia Appendix 1).

Four studies divided the participants into more than 2 groups. Lee et al [61] compared 3 groups: an MR intervention group (experimental group), a self-Otago exercise group (control group), and a yoga group. Phu et al [45] and Sápi et al [55] used an MR training group, a control group that performed physical exercises, and a control group that did not. Htut et al [47] divided participants into 4 groups: an MR-based exercise group, a brain exercise group, a control group that performed physical exercises, and a control group that did not. The remaining 17 studies divided the participants into 2 groups: experimental (MR intervention) and control groups.

Five studies [48,49,53,56,59] compared the effect of MR intervention with the results of a control group whose only intervention was in the form of educational materials and fall prevention recommendations. Eleven studies [28,29,44,50,52,54,57,58,60-62] compared the effects of MR intervention with those of conventional physical exercise. Three studies [45,47,55] with more than 2 groups of participants (see the above paragraph) compounded this comparison with the results of a control group that did not receive any physical intervention. Two studies [46,51] did not specify whether the control group performed any exercises during the experiment.

The majority of the studies performed the testing procedure before and after the intervention. Only 3 papers reported a follow-up study 6-9 months after the experiments [29,46,48], which allowed them to include the number or frequency of falls as an outcome measure.

**Table 2:** Summary of the qualitative results of the systematic review. Full table can be found in Multimedia Appendix 1.

Study	Type of exercise	Technology	Sample size (n); age (years)^a^ (experimental and control groups); gender	Duration of intervention and follow-up	Outcome measures
Park and Yim [60]	Virtual “kayaking”	3D VR^b^ program with a 3D beam projector	72; 72.97 (SD 2.98) and 74.11 (SD 2.88); male/female: 36/36	6 weeks: 2 times per week; 50 minutes per session	Cognitive function, muscle strength, standing and sitting balance
Mirelman et al [29]	Walking on a treadmill	VR system with a modified Microsoft Kinect, computer, and a large screen	302; 73.3 (SD 6.4) and 74.2 (SD 6.9); male/female: 182/100	6 weeks: 3 times per week; 45 minutes per session; follow-up after 6 months	Fall rate; gait speed; variability, endurance, balance; mobility; attention; and more
Phu et al [45]	Balance training	VR system (Balance Rehabilitation Unit)	195; median 78 (IQR 73–84); male/female: 65/130	6 weeks: 2 times per week; 30 minutes per session	Balance and physical performance, fear of falling
Yeşilyaprak et al [44]	Balance training	BTS NIRVANA VR Interactive System	18; 70.1 (SD 4.0) and 73.1 (SD 4.5); male/female: 6/12	6 weeks: 3 times per week; 50 minutes per session	Dynamic balance, standing balance, fall risk
Lee et al [61]	Otago exercises (exercises for leg muscle strength and balance, progressing in difficulty)	AR^c^-based Otago exercise	30; 72.60 (SD 2.67), 75.80 (SD 5.47), and 76.40 (SD 5.54); male/female: 0/30	12 weeks: 3 times per week; 60 minutes per session	Muscle strength, balance ability, static and dynamic load distribution, fall efficacy
Duque et al [46]	Balance training	VR system (Balance Rehabilitation Unit)	60; 79.3 (SD 10) and 75 (SD 8); male/female: 28/42 at baseline	6 weeks: 2 times per week; 20 minutes per session; follow-up after 9 months	Posture, fall rate, gait, grip strength, serum measurements, depression, fear of falling
Bacha et al [54]	Four Kinect Adventures games	Kinect Adventures Games for Microsoft Xbox 360 Kinect	46; mean 66.5 (range 65.0-71.75) and mean 71.0 (range 66.0-74.5); male/female: 12/34	7 weeks: 2 times per week; 60 minutes per session	Postural control, gait, cardiorespiratory fitness, cognition
Htut et al [47]	VR games and brain games	Microsoft Xbox 360 Kinect	84; 75.8 (SD 5.19); male/female: 47/37	8 weeks: 3 times per week; 30 minutes per session	Balance, muscle strength, cognition, fall concern
Gschwind et al [48]	Balance sessions and muscle strength sessions	iStoppFalls, PC/Kinect-based fall preventive exercise game (exergame)	153; 74.7 (SD 6.7) and 74.7 (SD 6.0); male/female: 60/93	16 weeks: 180 minutes per week; follow-up after 6 months	Fall risk, health status, health measures, physical measures, cognitive measures
Sápi et al [55]	Microsoft Xbox 360 Kinect videogames	Microsoft Xbox 360 Kinect	75; 69.57 (SD 4.66), 69.12 (SD 4.19), and 67.18 (SD 5.56); male/female: 6/70	6 weeks: 3 times per week; 30 minutes per session	Balance, reaction, movement velocity
Singh et al [58]	Wii Fit games	Nintendo Wii Fit with a Balance Board	36; 61.12 (SD 3.72) and 64.00 (SD 5.88); male/female: 0/36	6 weeks: 2 times per week; 60 minutes per session	Fall risk, fear of fall
Rendon et al [56]	Wii Fit games	Nintendo Wii Fit with a Balance Board (WBB)	40; 85.7 (SD 4.3) and 83.3 (SD 6.2); male/female: 14/26	6 weeks: 3 times per week; 35–45 minutes per session	Coordination and agility, dynamic balance, balance confidence
Stanmore et al [59]	Tailored fall prevention exergame program	Laptop and Kinect sensor	106; 77.8 (SD 10.2)/range 58-101 and 77.9 (SD 8.9)/range: 58-96; male/female: 23/83	12 weeks: total exercise time of 359 minutes; fall diary for 3 months after experiment	Balance, risk and fear of fall, mobility, pain, mood, fatigue, cognition, quality of life, falls
Yoo et al [62]	Otago exercise	Computer with a web camera, head-mounted display	21; 72.90 (SD 3.41) and 75.64 (SD 5.57); male/female: 0/21	12 weeks: 3 times per week; 80 minutes per session	Balance, gait, falls efficacy
Cho et al [49]	Wii Fit games	Nintendo Wii Fit with a Balance Board	32; 73.1 (SD 1.1) and 71.7 (SD 1.2); male/female: not specified	8 weeks: 3 times per week; 30 minutes per session	Balance
Ku et al [28]	Balance training	3D interactive system using a kinetic sensor	36; 64.7 (SD 7.27) and 65.0 (SD 4.77); male/female: 17/17	4 weeks: 3 times per week; 30 minutes per session	Dynamic balance, coordination, fall risk, posture
Anson et al [57]	Walking on a treadmill	TV, 2 webcams, stationary bulls-eye target	40; 75.8 (SD 6.5) and 75.7 (SD 5.3); male/female: 11/29	4 weeks: 3 times per week; 30 minutes per session	Gait, balance
Tsang and Fu [50]	Wii Fit games	Nintendo Wii Fit	79; 82.3 (SD 3.8) and 82.0 (SD 4.3); male/female: 31/48	6 weeks: 1 time per week; 60 minutes per session	Balance
Lee et al [51]	Wii Fit games	Nintendo Wii Fit with a Balance Board and Wii Fit joystick	40; 76.15 (SD 4.55) and 75.71 (SD 4.91); male/female: 17/23	6 weeks: 2 times per week; 60 minutes per session	Static and dynamic balance
Chen et al [52]	Traditional Tai Chi exercises	AR training system (based on Microsoft Kinect)	28; 72.2 (SD 2.8) and 75.1 (SD 5.5); male/female: 3/25	8 weeks: 3 times per week; 30 minutes per session	Balance
Jeon and Kim [53]	Regular, aerobic, and flexibility exercises	AR-based exercise rehabilitation system (UIN-HEALT); PC, a 3D motion analysis sensor	27; 72.77 (SD 3.79) and 72.71 (SD 3.64); male/female: 0/27	12 weeks: 5 times per week; 30 minutes per session	Physical performance, balance, mobility, walking skill, exercise, Self-Efficacy Scale

^a^Values (age) are listed as mean (SD) unless otherwise specified.

^b^VR: virtual reality.

^c^AR: augmented reality.

#### Duration of the Intervention

The duration of the intervention experiments in the selected studies ranged from 4 to 16 weeks. The frequency of the training in the majority of the studies (19 out of 21) was 2 to 3 exercise training sessions per week; the exceptions were the studies by Jeon and Kim [53], which had 5 training sessions per week, and Gschwind et al [48], which did not specify the number of training sessions per week but recommended 120 minutes/week for balance exergames and 60 minutes/week for strength exercises. The training sessions in all the studies had a duration of 30-60 minutes per session, except [48] which did not specify the duration of the training sessions.

#### Type of Exercise and Technology

As a physical exercise basis for the MR intervention, the studies used different conventional exercises; sets of training sessions and games, including kayaking; walking on a treadmill; variations of a balance training; and exercise complexes with different types of exercises. Seven studies used commercial console games: Kinect games [54,55] and Wii Fit games [49-51,56,58]. Two studies used walking on a treadmill [29,57]. Two used Otago exercises (exercises for leg muscle strength and balance, progressing in difficulty, in addition to a walking plan) [61,62]. Four studies used balance training [28,44-46]. Two used other sets of exercises: a combination of balance and muscle strength sessions [48] and regular, aerobic, and flexibility exercises [53]. Park and Yim [60] used virtual “kayaking,” Chen et al [52] used traditional Tai Chi exercises, Stanmore et al [59] used a tailored fall prevention exergame program, and Htut et al [47] used VR games for physical training and brain games for cognitive training.

Regarding the technology used in the selected studies, commercial off-the-shelf consoles were used in 7 of them: The Nintendo Wii Fit in 5 [49-51,56,58] and the Microsoft Xbox 360 Kinect in 2 [54,55]. Two studies used the Balance Rehabilitation Unit VR system [45,46]. Yeşilyaprak et al [44] used the BTS NIRVANA VR Interactive System, Jeon and Kim [53] used the AR exercise rehabilitation system UIN-HEALT, and Gschwind et al [48] used the iStoppFalls system, which is based on the Kinect sensor. Three additional studies used other systems based on the Kinect sensor [28,52,59].

#### Involvement of Health Care Professionals/Caregivers

Information about specialists’ involvement in exercise sessions was also extracted from the selected studies. Eight studies did not mention the involvement of any health care specialists or physiotherapists during the exercise training sessions. In 7 studies, the training sessions of the participants were supervised or lead by health care professionals [29,44,45,51,56,58,59]. In the study by Tsang and Fu [50], only control group training was led by health care professionals. In the study by Jeon and Kim [53], the intervention system was operated by an exercise specialist who controlled participant’s training modules and difficulty levels. In 4 studies, participants had 1 or several pilot sessions (trial sessions, familiarization sessions) [54,55,58,62]. In 6 studies participants received educational materials/booklets or fall prevention recommendations/instructions [46,48,51,57,59,61]. Participants in the study by Chen et al [52] received instructions from a certified Tai Chi master. In 2 studies, the exercises were conducted by health care specialists [47,55].

#### Outcome Measures and Results

The most common outcome measure was balance. Seventeen papers used balance (static and dynamic) as an outcome measure for their study, meaning only 4 studies [46,48,54,58] did not use balance as an outcome measure. Other outcome measures considered in the selected studies included gait parameters [29,54,57,62], muscle strength [47,60,61], mobility [29,53,59], and posture [28,46,54].

Four papers [29,46,48,59] evaluated not only fall risk factors but also the number of falls. For instance, Mirelman et al [29] demonstrated that the fall rate in the intervention group was significantly lower after the treatment than before. The results of the conventional exercise group did not show a significant decrease in the number of falls.

Eighteen studies demonstrated that the MR intervention had a positive effect on the fall risk factors and fall rate among elderly participants [28,29,45-53,55-57,59-62]. Three studies [44,54,58] did not find a significant difference between the experimental (MR intervention) and control groups.

### Quantitative Analysis: Principal Analysis Results

**Figure 4:**
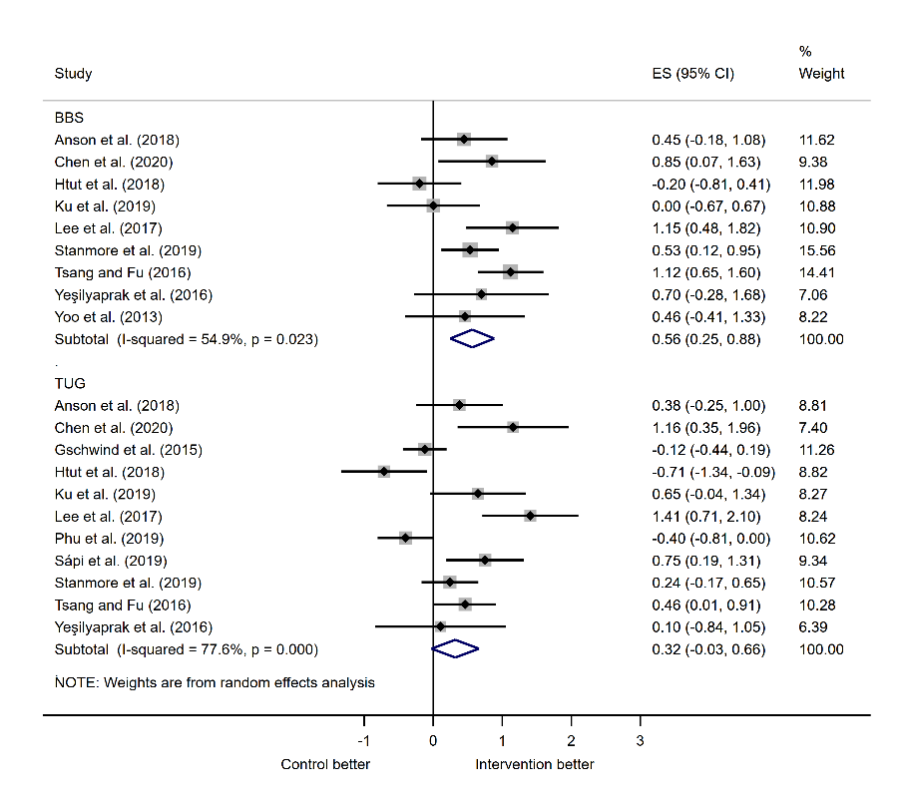
Meta-analysis forest plot [28, 44, 45, 47, 48, 50-52, 55, 57, 59, 62].

Data from BBS and TUG measures of physical function were pooled for quantitative analysis (Figure 4). Participants’ balance measured using the BBS was reported in 9 trials [28,44,47,50-52,57,59,62]. There was a statistically significant difference between the BBS scores of the intervention and control groups (ES: 0.564, 95% CI 0.246-0.882; *P*<.001) with heterogeneity statistics of *I*^2^=54.9% and *Q*=17.74 (*P*=.02). The robumeta analysis showed the overall pooling effect from the BBS and TUG measurements: the SMD was 0.37 (0.02-0.72), with *P*=.04.

Participants’ balance measured using the TUG was reported in 11 trials [28,44,45,47,48,50-52,55,57,59]. The analysis also showed a statistical difference between the TUG scores of the intervention and control groups, but this difference was not significant (ES: 0.318; 95% CI –0.025 to 0.662; *P*=.07). The heterogeneity statistics were *I*^2^=77.6% and *Q*=44.63 (*P*<.001).

**Figure 5:**
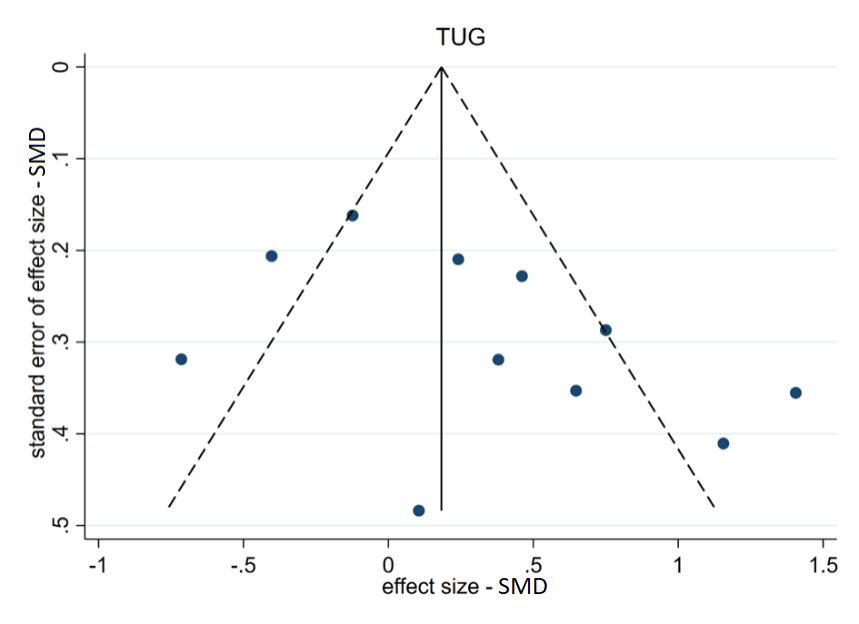
Funnel plot for TUG.

From the corresponding funnel plot (Figure 5) and the Egger test for small-study effects (*P*=.76 and *P*=.11) for the BBS and TUG scores, respectively, a minor publication bias in the studies on TUG (*P*=.11) might be present.

**Figure 6:**
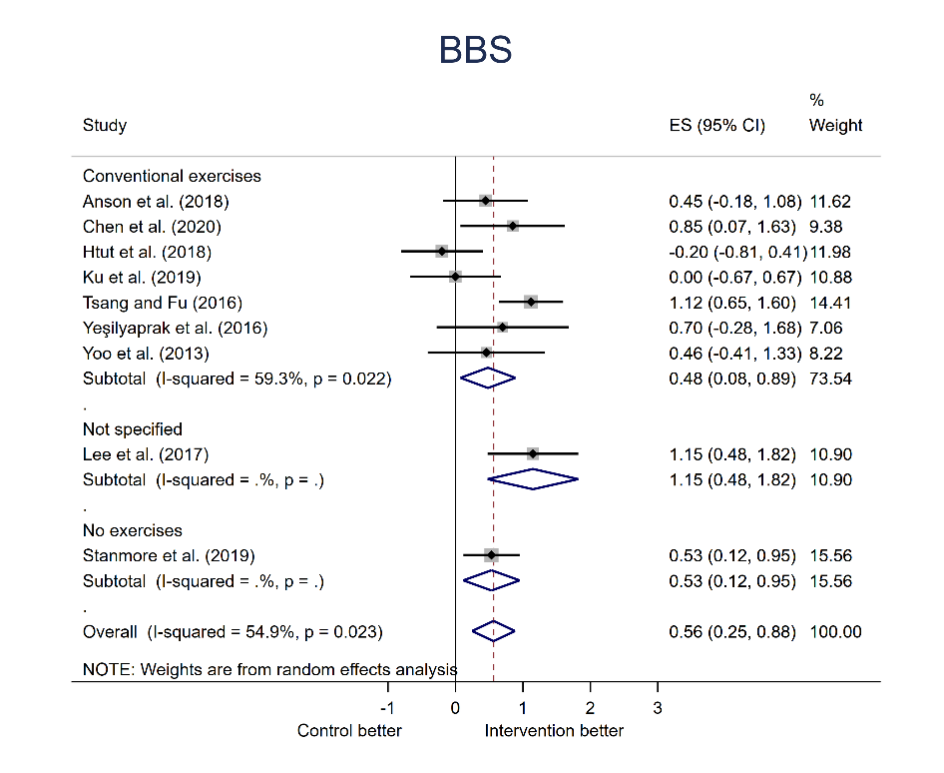
BBS test forest plot based on control group approach. BBS: Berg Balance Scale [28, 44, 47, 50-52, 57, 59, 62].

To explain the high level of heterogeneity, the data were assessed in relation to several different parameters: the control group approach (exercise/no exercise/not specified), the MR technology used (developed in-house/off-the-shelf product), and the total time participants spent exercising. However, no statistical differences were found among the groups. These results could be related to the small number of studies included in the meta-analysis, especially after they were split into several categories. For instance, there was only 1 study using the BBS test (Figure 6) that contained a control group that did not perform physical exercise. Alternatively, the high level of heterogeneity could be related to other differences in study design or participant demographics, such as differences in mean participant age or exercise intensity.

Overall, both BBS and TUG results demonstrate improvement with the MR intervention. The BBS improvement was found to be significant with a bias (*P*<.001), whereas the TUG improvement was not significant (*P*=.07).

## Discussion

### Principal Findings

The systematic literature review provides an analysis of MR technologies that have been used for elderly training and use of MR interventions in the studies (RQ1). The results showed a common use of commercial consoles and a lack of specialized software/hardware that focuses on physical training and developed specifically for older users. The study also revealed a positive effect of MR intervention on the fall risk factors of older adults. The quantitative analysis of BBS and TUG shows an improvement of participants’ physical functions (RQ2).

### Number of Relevant Papers

Although our database search yielded a high number of candidate articles, only 21 studies satisfied all inclusion and exclusion criteria and were included in this qualitative analysis. Because these studies used a wide variety of outcome measures, only 12 were included in the quantitative meta-analysis. Many papers were excluded because their research focus was outside the scope of this work, for example, focusing on a particular disease, investigating fear of falling, or considering participants’ emotions and attitudes toward MR. This indicates the huge scope of current research interests in MR technologies for older adults; however, it also reveals a dearth of studies focused on the effects of MR intervention on the physical health and risk of falling of healthy older individuals. According to Hamm et al [63], technological interventions (including MR) have been developed for various purposes within the scope of fall prevention research, such as diagnostics, fall detection, self-assessment for assistive equipment or fall risk treatment, postfall treatment, and injury prevention. This could explain the high number of initial search results, because, despite their different focuses, all such studies consider technical intervention in the context of falls with older adults as a target group. However, in this study, we focused only on studies that considered the effect of MR fall prevention interventions on health-related fall risk factors and fall frequency.

### Study Design

The selected studies had substantial variations in study design and intervention protocols. For instance, the studies had different durations and numbers of physical training sessions per week; the total exercise time in the studies included in the meta-analysis spanned a wide range, from 360 to 2880 minutes. Additionally, the studies used different types of physical activities and investigated different MR technologies for intervention, and the papers included little to no information about the training intensity. The number of participants and their demographics also differed from one study to another. All of these factors complicate the process of systematically comparing the studies and their outcomes.

One substantial point of variation among the study designs was the control group approach. In several studies [28,29,44,50,52,54,57,58,60-62], control groups performed traditional physical training, whereas in other studies [48,49,53,56,59], control groups did not do any physical exercise, and some studies did not specify this aspect at all. The control group approach is crucial in understanding the effects of interventional MR technologies. If a study compares an MR intervention group with a group that did not perform any physical exercise, the effect cannot be explicitly attributed to the MR intervention, because the MR and the physical exercise are likely to have produced a combined effect. Because the focus of this research is on the effect of MR intervention on the training process, the studies that compare traditional intervention methods with intervention using MR technology have more relevance in the context of our research.

The diversity of the study design, participant demographics, sample sizes, and intervention protocols makes it difficult to compare the studies and their outcomes. Choi et al [64] came to a similar conclusion in their systematic review of exergames and interactive interventions for elderly fall prevention. They claimed that there was no consensus regarding study design among the papers they included in their review, which made it difficult to draw scientific conclusions on the effectiveness of different intervention methodologies.

Therefore, it is important to develop general design recommendations for studies aimed at the assessment of the effectiveness of technical intervention as a fall prevention method for older adults. In addition, more studies with large sample sizes should be performed based on the developed recommendations to allow the comparison of the results across studies and the formulation of scientific conclusions on the effectiveness of MR intervention.

### Outcome Measures

The selected studies used different outcome measures, which made it complicated to compare the results across studies and perform a meta-analysis. The studies used different physical and cognitive parameters, such as the participants’ static and dynamic balance, to represent the outcome of the experiment. Furthermore, they used different tests and test systems, such as the BBS and TUG scores for dynamic balance assessment, to quantify these parameters. Eleven studies used the TUG to measure the dynamic balance of the participants as a primary or secondary outcome measure, and 9 studies used the BBS for the same purpose. However, dynamic balance is just one of many common physical parameters that are commonly accepted as an indicator of fall risk among the elderly. The considered studies also used gait, muscle straight, and mobility as the outcome measures, and some included cognitive function tests or fear of falling assessment.

In addition to complicating the comparison and analysis of the results, the differences in outcome measures indicate that there is no consensus on which parameters are important to consider in research on fall prevention in older adults. According to Perell et al [65], fall risk assessment does not have a standard within or across settings. Choi et al [64] mentioned in their review that there was a wide variety of outcome measures in the reviewed publications. Therefore, more studies using the same outcome measures should be performed to enable a comparison of their results.

Three studies [29,46,48] included a follow-up 6-9 months after intervention, and in another study [59] authors instructed participants to keep a 3-month fall diary; both of these approaches made it possible to investigate the actual rate of falls experienced by older adults after they participated in the studies. Follow-up studies also allow researchers to measure study outcomes a long time after the experiment and determine if the participant continues exercising and maintains the results of the training. This review demonstrates a lack of longitudinal studies and studies with a follow-up period on the order of months on MR interventions for fall prevention. Future research in this area should focus on longitudinal studies.

In the context of fall rate measurement, it is also important to mention that only 4 studies [29,44-46] collected information on the participants’ previous falls history and the remaining 17 papers did not specify the participants’ fall history or if they were at a risk of falling. However, current history of falls is a fall risk factor [9] and could influence the results of the data analysis, especially in the case of fall rate assessment. Therefore, it is recommended that information about participants’ previous history of falls and fall risks be collected in MR fall prevention intervention research.

### Involvement of Health Care Personnel/Caregivers and Introduction to Participants

One of the aspects we wanted to investigate was the involvement of health care specialists or caregivers in experiments process. In several studies, the training sessions of the participants (experimental group or control group) were supervised or led by health care professionals [29,44,45,50,51,53,56,58,59]. However, the papers do not provide many details on how the training supervision was performed or how much assistance the participants needed from the health care professionals. Moreover, 8 studies did not mention the involvement of any health care specialists or physiotherapists during the exercise training sessions [29,44,45,50,51,56,58,59].

In several studies, the participants also received information, recommendations, or instructions on the use of MR technology and how to perform the assigned physical exercises in different forms. However, the corresponding publications did not mention how much MR assistance the participants needed during the experiments or how much help the health care specialists and physiotherapists provided during the MR intervention. This makes it difficult to understand if the results of the training could differ depending on each user’s ability to use the MR technology unassisted.

### Factors That Can Influence the Outcome of the MR Intervention

MR intervention is treated as a black box in most of the studies. There are many factors that can affect the outcome. For instance, the papers did not provide any details on the usability issues that elderly participants may have experienced with the MR intervention. According to the literature, elderly users have high demands regarding the usability of technology [66] and tend to have low acceptance of technology [67]. According to Tuena et al [68], usability is very important, especially for virtual systems used in medical contexts. Usability influences the ease with which a task can be accomplished, whether the user can perform the task quickly, whether the user can retain an understanding of how to use the system, and how many errors the user makes [68]. We can assume that all of these parameters influence the effect of the MR-based physical activity. For these reasons, it is important to understand if participants have experienced any usability issues during the intervention.

In addition to usability there are other factors that can influence the acceptance of technology by elderly. Because an evaluation of the acceptance of technology was not a priority of this review and meta-analysis, we excluded studies that focused specifically on acceptance and perception aspects of MR intervention technology for fall prevention. However, considering such factors including usability as an additional characteristic in the context of physical function testing could reveal confounding factors that could influence the results of the studies.

### Variety of Type of Training Sessions and Technologies

Different types of physical activities were used in the selected studies, including kayaking, variations of a balance training, and Tai Chi. The complexity of the exercises ranged from a simple walk on a treadmill to a complete exercise program including several types of exercises such as balance training and strength exercises. However, despite the difference in the exercise approach, all the studies demonstrated that MR intervention had a positive effect on the physical functions of the participants. From this, we may conclude that the mere *performance* of regular physical exercise has a greater value and importance than the *type* of exercise. Consequently, the future development of MR systems for fall prevention should focus on users’ motivation to exercise regularly. However, very few studies used multiple types of physical activities. To perform a meta-analysis and investigate possible differences in effectiveness among various types of exercises, more studies that use the same type of physical activity and the same study design would be necessary. Thus, further investigation of MR interventions in combination with different physical activities is needed to determine the most effective combination for fall prevention among older adults.

Regarding the type of MR technology, 7 studies [49-51,54-56,58] used commercial video game consoles (Microsoft Kinect motion sensor device and Nintendo Wii video game console). The results of this review demonstrate a lack of specialized software/hardware developed for physical exercises with older users as a target group. Future research should focus on developing and testing MR fall prevention systems designed specifically for older users that would, for instance, help to increase usability within the target group.

### Terminology

One of the main challenges we experienced during the review process is the inconsistent use of terminology in defining MR, VR, and AR technologies. Some of the studies that used similar interventions or sometimes even the same system used different terms to define it. For example, in the study by Ku et al [28], a Microsoft Kinect, a laptop, and a big screen were used to create an interactive training environment, which the authors called an AR system, whereas Mirelman et al [29] used a modified Microsoft Kinect, a computer, and a large screen to create a very similar set up but described it as a VR. This clash in terminology despite the similarity of the systems demonstrates the need for further investigation of MR interventions and clearer definitions of the terms AR and VR, especially because of how popular these technologies have become. Clearer definitions would help researchers in their investigation of the benefits of these technologies in relation to fall prevention among older adults.

### Limitations

This review was limited by the high degree of heterogeneity in the data pooled for meta-analysis. Although the evaluated studies share similar aims and methodologies, there were many differences regarding their study design and outcome measures, such as experiment duration, types of physical exercises and technologies, and participant demographics. We acknowledge the high statistical heterogeneity across the meta-analysis indicating unknown between-trial variability. The results of the meta-analysis should therefore be viewed with caution.

Another limitation of this study is that we did not assess quality of the selected studies, for instance, using instruments such as GRADE (Grading of Recommendations, Assessment, Development and Evaluations) [69] or the PEDro scale (The Physiotherapy Evidence Database) [70]. However, a thorough qualitative analysis of the studies was performed.

In this systematic review, we considered studies that included an investigation of the effect of MR intervention against falls among older adults, which is measured as the number of falls or risk factors for falling. However, we excluded publications that focused only on fear of falling, falls self-efficacy, and cognitive functions, although these are also fall risk factors. Further research should investigate MR fall prevention interventions in relation to these outcome measures.

### Recommendations for Future Work

MR technology has the potential to help prevent falls among the elderly and improve the overall quality of their lives. However, more research should focus on the effect of MR intervention on elderly physical health and the risk of falling among healthy elderly individuals. More specifically, we find that the following main points should be addressed in future work.

- General recommendations regarding study design and intervention protocols should be developed for MR fall prevention intervention research.
- More research should be conducted with a homogeneous study design and using the same outcome measures.
- More longitudinal studies on MR fall prevention interventions should be conducted.
- Researchers should collect information on participants’ history of falls.
- The involvement of health specialists and caregivers in experiments should be specified.
- More studies on the effects of MR fall prevention intervention should also include a parallel study on factors such as MR usability that can influence the acceptance of the technology and the outcome of the intervention.

### Conclusion

The systematic review and meta-analysis were performed to gather data and summarize evidence of the effectiveness of MR interventions as a fall prevention method for older adults. To our knowledge, this is the first systematic review and meta-analysis to examine the effectiveness of the full spectrum of MR technologies for fall prevention intervention. Previous similar reviews have focused specifically on VR [27] and exergames [64].

The qualitative analysis on the selected papers demonstrated that results of most studies showed the positive effect of MR intervention on the fall risk factors of older participants. The meta-analysis results suggest that MR interventions improved physical function as measured by the BBS and TUG scores. The results of this study are in agreement with a recent systematic literature review [64] on the use of exergames for fall prevention and a recent meta-analysis [27] on the effect of VR interventions on mobility and balance among older adults.

This study demonstrated that the use of MR interventions has a positive effect on physical functions in older adults. MR has the ability to help elderly users perform physical exercises, which could prevent falls among older adults and improve their health conditions. More research should focus on the effect of MR interventions on elderly health and the risk of falling among healthy elderly individuals.

## Appendix

Multimedia Appendix 1 Summary of the qualitative results of the systematic review.

## Abbreviations

5TSTS: 5 Times Sit to Stand
ABC: Activity-Specific Balance Confidence Scale
AR: augmented reality
BBS: Berg Balance Scale
BESTest: Balance Evaluation Systems Test
GBS: Good Balance System
MR: mixed reality
SMD: standardized mean difference
TUG: Timed Up and Go
VR: virtual reality
