# Supplementary material for: The Effect of Mixed Reality Technologies for Falls Prevention Among Older Adults: Systematic Review and Meta-analysis
Source: JMIR Aging. 2021 Jun 30;4(2):e27972. doi: 10.2196/27972 (PMC8280833; doi:10.2196/27972)
Supplement: Multimedia Appendix 1 [file aging_v4i2e27972_app1.pdf]

	Study	Aim	Type of exercise	Technology	Sample size, age (years) (experimental and control groups), gender	Participants' history or risk of falls	Study design	Involvement of health care professionals	Duration of intervention and follow-up	Outcome measures	Measurements (of physical functions)	Results after intervention
1	Park and Yim [60]	Investigate if a VR[a] kayak program could improve cognitive function, muscle strength, balance	Virtual "kayaking"	3D VR program with a 3D beam projector	72; 72.97 (SD 2.98) and 74.11 (SD 2.88); male/female: 36/36	No mention	Randomized controlled trial	Not specified	6 weeks: 2 times per week; 50 minutes per session	Cognitive function, muscle strength, standing and sitting balance	Handgrip strength, arm curl test, GBS[e]	Cognitive function (*P*<.05), muscle strength (*P*<.05), and balance (*P*<.05) were significantly improved in EG compared with CG.
2	Mirelman et al [29]	Test if a combination of treadmill training with VR leads to fewer falls than treadmill training alone	Walking on a treadmill	VR system with a modified Microsoft Kinect, computer, and a large screen	302; 73.3 (SD 6.4) and 74.2 (SD 6.9); male/female: 182/100	Experienced fall	Randomized controlled trial	Trainer present at all training sessions	6 weeks: 3 times per week; 45 minutes per session; follow-up after 6 months	Fall rate; gait speed; variability, endurance, balance; mobility; attention; and more	Zeno instrumented walkway and PKMAS, 2MWT[f], Short Physical Performance Battery	Incident rate after training was significantly lower in EG than before training; incident rate did not decrease significantly in CG.
3	Phu et al [45]	Compare the effects of VR training with the Balance Rehabilitation Unit VS exercise using a modified Otago Exercise Program on balance and physical performance	Balance training	VR system (Balance Rehabilitation Unit)	195; median 78 (IQR 73–84); male/female: 65/130	Experienced fall or balance issues	Intervention and control group (participants were not randomized)	Sessions were conducted and supervised by an accredited exercise physiologist	6 weeks: 2 times per week; 30 minutes per session	Balance and physical performance, fear of falling	5TSTS[g], TUG[h], gait speed and posturography assessment using the BRU, Falls Efficacy Scale, Handgrip strength and adherence	EG and CG achieved similar improvements in balance and physical performance measures. Only EG improved control of static posture in eyes closed (*P*=.002) and foam eyes closed (*P*=.006) tasks.
4	Yeşilyaprak et al [44]	Investigate the effects of VR-based balance exercises on balance and fall risk in comparison with conventional balance exercises	Balance training	BTS NIRVANA VR Interactive System	18; 70.1 (SD 4.0) and 73.1 (SD 4.5); male/female: 6/12	Experienced fall	Intervention and control groups	Supervised by a trained physiotherapist with more than 2 years of experience with the VR system	6 weeks: 3 times per week; 50 minutes per session	Dynamic balance, standing balance, fall risk	BBS[i], TUG, left leg stance and tandem stance duration with eyes closed	Balance and fall risk significantly improved with time (*P*<.05), but changes were similar in EG and CG (*P*>.05).

	Study	Aim	Type of exercise	Technology	Sample size, age (years) (experimental and control groups), gender	Participants' history or risk of falls	Study design	Involvement of health care professionals	Duration of intervention and follow-up	Outcome measures	Measurements (of physical functions)	Results after intervention
5	Lee et al [61]	Determine the effect of AR[b]-based Otago exercise on muscle strength, balance, and physical factors in older women	Otago exercises (exercises for leg muscle strength and balance, progressing in difficulty)	AR-based Otago exercise	30; 72.60 (SD 2.67), 75.80 (SD 5.47), and 76.40 (SD 5.54); male/female: 0/30	No mention of fall risk/rate	Randomized controlled trial	Not specified. Fall prevention education was provided to participants once	12 weeks: 3 times per week; 60 minutes per session	Muscle strength, balance ability, static and dynamic load distribution, fall efficacy	MMT[j], FP[k], MFS[l]	Knee flexion and ankle dorsiflexion strength significantly improved in all groups. Balance, measured as eye open center of pressure (CoP)-x, significantly decreased in the EG and yoga groups. Eye closed CoP-x, eye open standard deviation-x, and eye open height of ellipse significantly decreased only in EG. EG showed improvement in MFS.
6	Duque et al [46]	Evaluate the effect of a VR system (Balance Rehabilitation Unit) on balance, falls, and fear of falling	Balance training	VR system (Balance Rehabilitation Unit)	60; 79.3 (SD 10) and 75 (SD 8); male/female: 28/42 at baseline	Experienced fall or balance issues	Intervention and control groups	Not specified. Groups received fall prevention recommendations and education	6 weeks: 2 times per week; 20 minutes per session; follow-up after 9 months	Posture, fall rate, gait, grip strength, serum measurements, depression, fear of falling	Fall rate, gait rate, Groningen Elderly Test, balance parameters	EG showed significant improvement in balance parameters (*P*<.01), significant reduction in falls, and lower levels of fear of falling (*P*<.01). Some components of balance that were improved in EG showed a decline 9 months after training.
7	Bacha et al [54]	Compare the effectiveness of Kinect Adventures games versus conventional physiotherapy to improve postural control, gait, cardiorespiratory fitness, and cognition	Four Kinect Adventures games	Kinect Adventures Games for Microsoft Xbox 360 Kinect	46; mean 66.5 (range 65.0-71.75) and mean 71.0 (range 66.0-74.5); male/female: 12/34	No mention of fall risk/rate	Randomized clinical trial	Familiarization sessions with a therapist's instructions and explanation. Participants who passed were excluded	7 weeks: 2 times per week; 60 minutes per session	Postural control, gait, cardiorespiratory fitness, cognition	Mini-BESTest[m], Functional Gait Assessment, 6MST[n]	No significant differences between EG and CG; both interventions provided positive effects on postural control, gait, cardiorespiratory fitness, and cognition (post hoc Tukey test; *P*<.05).

	Study	Aim	Type of exercise	Technology	Sample size, age (years) (experimental and control groups), gender	Participants' history or risk of falls	Study design	Involvement of health care professionals	Duration of intervention and follow-up	Outcome measures	Measurements (of physical functions)	Results after intervention
8	Htut et al [47]	Compare the effects of physical exercise, VR-based exercise, and brain exercise on balance, muscle strength, cognition, and fall concern	VR games and brain games	Microsoft Xbox 360 Kinect	84; 75.8 (SD 5.19); male/female: 47/37	No mention of fall risk/rate	Randomized controlled trial	Exercises were conducted by a physiotherapist	8 weeks: 3 times per week; 30 minutes per session	Balance, muscle strength, cognition, fall concern	BBS, TUG, 5TSTS, left and right HGS[o]	CG showed significant improvement in TUG and 5TSTS to a greater extent than EG (TUG: *P*=.004, 5TSTS: *P*=.02). EG significantly improved MoCA (*P*<.001) and FES-I (*P*=.03) compared with CG. EG and CG significantly (*P*<.001) increased Borg CR-10 in all exercise sessions. EG reported higher enjoyment than CG (*P*<.001).
9	Gschwind et al [48]	Assess the feasibility (exercise adherence, acceptability, and safety) of the intervention program and its effectiveness against common fall risk factors	Balance sessions and muscle strength sessions	iStoppFalls, PC/Kinect-based fall preventive exercise game (exergame)	153; 74.7 (SD 6.7) and 74.7 (SD 6.0); male/female: 60/93	No mention of fall risk/rate	Randomized controlled trial	Not specified. Groups received educational booklets	16 weeks: 180 minutes per week; follow-up after 6 months	Fall risk, health status, health measures, physical measures, cognitive measures	Physiological Profile Assessment, 12-item WHO Disability Assessment Schedule, tests for coordinated stability and maximal balance range, static balance, walking speed over 4 m, 5TSTS, TUG	Physiological fall risk reduced significantly more in EG compared with CG ($F_{1,127}$=4.54, *P*=.03).
10	Sápi et al [55]	Find out if Kinect training is superior to conventional balance training in aspects of functional balance tests and posturography measurements testing postural stability through visual feedback	Microsoft Xbox 360 Kinect videogames	Microsoft Xbox 360 Kinect	75; 69.57 (SD 4.66), 69.12 (SD 4.19), and 67.18 (SD 5.56); male/female: 6/70	No mention of fall risk/rate	Intervention, conventional exercise, and control groups	The training sessions were conducted by physiotherapists. Pilot testing session	6 weeks: 3 times per week; 30 minutes per session	Balance, reaction, movement velocity	FSST[p], Functional Reach Test; TUG, LOS[q]	EG and CG showed progress in the follow-up measurements. More statistically significant improvements were found in EG in TUG (*P*<.05), TUG cognitive dual-task test (*P*<.05), Four-Square Step Test (*P*<.05), Functional Reach Test (*P*<.05), LOS movement velocity (*P*<.05).

	Study	Aim	Type of exercise	Technology	Sample size, age (years) (experimental and control groups), gender	Participants' history or risk of falls	Study design	Involvement of health care professionals	Duration of intervention and follow-up	Outcome measures	Measurements (of physical functions)	Results after intervention
11	Singh et al [58]	Quantify the effectiveness of VR balance games in decreasing risk and fear of falls among older women	Wii Fit games	Nintendo Wii Fit with a Balance Board	36; 61.12 (SD 3.72) and 64.00 (SD 5.88); male/female: 0/36	No mention of fall risk/rate	Randomized controlled trial	Demonstration and trial session were provided. Participants performed the games on their own with one-on-one supervision by a therapist assistant	6 weeks: 2 times per week; 60 minutes per session	Fall risk, fear of fall	Physiological Profile Approach, ABC[r]	Both EG and CG showed a significant decrease in PPA (*P*<.001) and ABC-6 (*P*<.01). No significant effects were demonstrated between the groups in PPA (*P*=.18) and ABC-6 (*P*=.25). Time and group interaction effect were not significant for PPA (*P*=.18) and ABC-6 (*P*=.45).
12	Rendon et al [56]	Determine the feasibility and outcome using the Wii Fit games for improvement of dynamic balance	Wii Fit games	Nintendo Wii Fit with a Balance Board (WBB)	40; 85.7 (SD 4.3) and 83.3 (SD 6.2); male/female: 14/26	No mention of fall risk/rate	Randomized clinical trial	For EG[c], a physical therapist was present to assist the participant in the operation of the game or in stepping on or off the WBB	6 weeks: 3 times per week; 35–45 minutes per session	Coordination and agility, dynamic balance, balance confidence	8 Foot Up and Go, ABC, Geriatric Depression Scale	Compared with CG, EG showed significant improvements in 8 Foot Up and Go (median decrease of 1.0 seconds vs −0.2 seconds, [*P*=.03] and ABC [6.9% vs 1.3%, *P*=.03]).
13	Stanmore et al [59]	Determine the effectiveness of a tailored OTAGO/FaME-based strength and balance exergame for reducing falls risk	Tailored fall prevention exergame program	Laptop and Kinect sensor	106; 77.8 (SD 10.2)/range 58-101 and 77.9 (SD 8.9)/range: 58-96; male/female: 23/83	No mention of fall risk/rate	Randomized controlled trial	Supported by physiotherapists or trained assistants. Educational materials	12 weeks: total exercise time of 359 minutes; fall diary for 3 months after experiment	Balance, risk and fear of fall, mobility, pain, mood, fatigue, cognition, quality of life, falls	BBS, fall rate (diary), TUG, Fall risk score, Physical Activity Scale for the Elderly	There was an adjusted mean improvement in balance (BBS) of 6.2 (95% CI 2.4-10.0) and reduced fear of falling (*P*=.007) and pain (*P*=.02) in EG. The change in fall rates significantly favored the EG (incident rate ratio 0.31 [95% CI 0.16-0.62, *P*=.001]).
14	Yoo et al [62]	Determine the effects of AR-based Otago exercise on balance, gait, and falls efficacy of older women.	Otago exercise	Computer with a web camera, head-mounted display	21; 72.90 (SD 3.41) and 75.64 (SD 5.57); male/female: 0/21	No mention of fall risk/rate	Intervention and control groups	Not specified. The first week started with a moderate-intensity exercise program	12 weeks: 3 times per week; 80 minutes per session	Balance, gait, falls efficacy	BBS, gait parameters (velocity, cadence, step length, stride length), falls efficacy	EG showed significant increases in BBS, velocity, cadence, step length (right side), stride length (right side and left side), and falls efficacy.

	Study	Aim	Type of exercise	Technology	Sample size, age (years) (experimental and control groups), gender	Participants' history or risk of falls	Study design	Involvement of health care professionals	Duration of intervention and follow-up	Outcome measures	Measurements (of physical functions)	Results after intervention
15	Cho et al [49]	Determine the effects of VR-based balance training on balance	Wii Fit games	Nintendo Wii Fit with a Balance Board	32; 73.1 (SD 1.1) and 71.7 (SD 1.2); male/female: not specified	No mention of fall risk/rate	Intervention and control groups	Not specified	8 weeks: 3 times per week; 30 minutes per session	Balance	Romberg test on a Bio-rescue	Body center of pressure movement area of EG with eyes open and closed significantly improved (*P*<.05), while CG did not (*P*>.05).
16	Ku et al [28]	Evaluate the clinical efficiency of a novel 3D interactive AR system (3D-ARS) for balance and mobility rehabilitation	Balance training	3D interactive system using a kinetic sensor	36; 64.7 (SD 7.27) and 65.0 (SD 4.77); male/female: 17/17	No mention of fall risk/rate	Randomized controlled trial	Not specified	4 weeks: 3 times per week; 30 minutes per session	Dynamic balance, coordination, fall risk, posture	BBS, TUG, functional ambulation category, modified Barthel index; lower extremity subscale of the Fugl-Meyer Assessment, Fugl-Meyer motor Assessment, automatic balance score	Both groups showed improvements in stability index, weight distribution index, fall risk index, and Fourier transformation index after urography. Changes were significantly greater in EG.
17	Anson et al [57]	Determine whether trunk motion VFB treadmill walking would improve over-ground balance	Walking on a treadmill	TV, 2 webcams, stationary bulls-eye target	40; 75.8 (SD 6.5) and 75.7 (SD 5.3); male/female: 11/29	No mention of fall risk/rate	Intervention and control groups	Not specified. Participants were instructed	4 weeks: 3 times per week; 30 minutes per session	Gait, balance	The BESTest, mini-BESTest, BBS, TUG, ABC, 6MWT	EG significantly improved on the BESTest (*P*=.03) and the mini-BEST (*P*=.01). CG did not improve significantly on any measure.
18	Tsang and Fu [50]	Compare Wii Fit balance training with conventional balance training	Wii Fit games	Nintendo Wii Fit	79; 82.3 (SD 3.8) and 82.0 (SD 4.3); male/female: 31/48	No mention of fall risk/rate	Randomized clinical trial	CG[d] training regimen was led by a physiotherapist	6 weeks: 1 time per week; 60 minutes per session	Balance	BBS, TUG, endpoint and maximum excursions in all 4 directions in the limits of stability test	BBS score, TUG, endpoint and maximum excursions in all 4 directions in the limits of stability test improved significantly in EG. CG significantly improved only BBS and TUG.

	Study	Aim	Type of exercise	Technology	Sample size, age (years) (experimental and control groups), gender	Participants' history or risk of falls	Study design	Involvement of health care professionals	Duration of intervention and follow-up	Outcome measures	Measurements (of physical functions)	Results after intervention
19	Lee et al [51]	Use 3D video gaming technology in VR training to improve postural balance and lower extremity strength	Wii Fit games	Nintendo Wii Fit with a Balance Board and Wii Fit joystick	40; 76.15 (SD 4.55) and 75.71 (SD 4.91); male/female: 17/23	No mention of fall risk/rate	Intervention and control groups	Six assistants monitored participants throughout the program. Groups received fall prevention education	6 weeks: 2 times per week; 60 minutes per session	Static and dynamic balance	GBS, 1-legged stance test, BBS Functional Reach Test, TUG, sit-to-stand test	EG showed significant improvements in static and dynamic postural balance and lower extremity strength (*P*<.05). EG improved overall in the considered parameters compared with CG (*P*<.05).
20	Chen et al [52]	Compare the beneficial effects of AR-assisted selected Tai Chi intervention with the complete sequence of traditional Tai Chi exercises	Traditional Tai Chi exercises	AR training system (based on Microsoft Kinect)	28; 72.2 (SD 2.8) and 75.1 (SD 5.5); male/female: 3/25	No mention of fall risk/rate	Prospective randomized trial	CG received instruction from a certified Tai-Chi master	8 weeks: 3 times per week; 30 minutes per session	Balance	BBS, TUG, Functional Reach Test, muscle strength measurements of the lower extremities	EG showed significant improvement in BBS, TUG, and Functional Reach Test. Although all 3 functional balance test scores improved in CG, only the improvement in BBS was statistically significant (*P*<.001). All muscle strength measurements increased by an average of 3.1 ± 1.0 kgw in EG and 1.6 ± 0.8 kgw in CG.
21	Jeon and Kim [53]	Prevent rapidly decreasing muscle loss in the elderly and confirm the effect of AR-based exercise on the exercise self-efficacy in the elderly, thus exploring the sustainability of AR-based exercise	Regular, aerobic, and flexibility exercises	AR-based exercise rehabilitation system (UIN-HEALT); PC, a 3D motion analysis sensor	27; 72.77 (SD 3.79) and 72.71 (SD 3.64); Male/female: 0/27	No mention of fall risk/rate	Intervention and control groups	AR system was operated by an exercise specialist who controlled the participants' training modules and difficulty levels	12 weeks: 5 times per week; 30 minutes per session	Physical performance, balance, mobility, walking skill, exercise, Self-Efficacy Scale	Appendicular skeletal muscle mass, skeletal muscle index, gait speed, senior fitness test, 2MST[s], TUG	Appendicular skeletal muscle mass (*F*=11.222, *P*<.002) and skeletal muscle index (*F*=11.222, *P*<.002) increased more in EG than in CG (*F*=11.222, *P*<.002); gait speed was significantly increased (*F*=7.221, *P*<.005). Exercise self-efficacy was significantly increased in EG (*F*=20.464, *P*<.001) compared with CG.

[a]VR: virtual reality.

[b]AR: augmented reality.

[c]EG: experimental group.

[d]CG: control group.

[e]GBS: good balance system.

[f]2MWT: 2-Minute Walk Test.

[g]5TSTS: 5 Times Sit to Stand.

[h]TUG: Timed Up and Go.

[i]BBS: Berg Balance Scale.

[j]MMT: Manual Muscle Test.

[k]FP: force plate.

[l]MFS: Morse Fall Scale.

[m]BESTest: Balance Evaluation Systems Test.

[n]6MST: 6-Minute Step Test.

[o]HGS: hand grip strength.

[p]FSST: The Four Square Step Test.

[q]LOS: limits of stability.

[r]ABC: Activity-Specific Balance Confidence Scale.

[s]2MST: 2-Minute Step Test.
